# Childhood maltreatment and alcohol and tobacco use trajectories in rural Chinese adolescents

**DOI:** 10.1186/s13034-024-00744-w

**Published:** 2024-05-03

**Authors:** Li Niu, Doran C. French, Yuyan Wang, Jianing Sun, Danhua Lin

**Affiliations:** 1 Faculty of Psychology, Beijing Normal University, Beijing, China; 2 Department of Human Development and Family Science, Purdue University, West Lafayette, IN USA; 3 Institute of Developmental Psychology, Faculty of Psychology, Beijing Normal University, 19 XinJieKouWai St., Haidian District, Beijing, China

**Keywords:** Sexual abuse, Physical abuse, Neglect, Substance use, Adolescence, School and neighborhood environment

## Abstract

**Background:**

There is a high prevalence of childhood maltreatment among Chinese children and adolescents, but little is known about its impact on alcohol and tobacco use trajectories and how positive school and neighborhood environments moderate the associations. The objective of this study was to assess the association between multiple forms of childhood maltreatment and longitudinal alcohol and tobacco use trajectories, and to assess the possibility that perceived connections to school and neighborhood moderate these associations.

**Methods:**

This longitudinal cohort study included 2594 adolescents (9 to 13 years) from a low-income rural area in China. Childhood exposure to abuse and neglect was assessed using the Childhood Trauma Questionnaire. Participants reported past-month alcohol and tobacco use at three time points over 1 year.

**Results:**

Growth curve models revealed that childhood sexual abuse was associated with a higher risk of past-month drinking (OR = 1.53, 95% CI 1.19–2.03, *p* < 0.001) and smoking (OR = 1.82, 95% CI 1.30–2.55, *p* < 0.001). Neglect was associated with a higher risk of past-month drinking (OR = 1.52, 95% CI 1.06–1.90, *p* < 0.05) and smoking (OR = 2.02, 95% CI 1.34–3.02, *p* < 0.001). None of the maltreatment forms predicted a faster increase in either drinking or smoking. These associations were found independent of personal, family, and contextual characteristics. School and neighborhood connection moderated the association between physical abuse and past-month drinking, such that physical abuse was associated with a greater risk of drinking only for youth who perceived low school or neighborhood connections.

**Conclusions:**

Findings demonstrate the importance of early experiences of childhood maltreatment for adolescent alcohol and tobacco use. Enhancing school and neighborhood connectedness for physically abused youth may help protect them from alcohol use.

**Supplementary Information:**

The online version contains supplementary material available at 10.1186/s13034-024-00744-w.

## Introduction

Childhood maltreatment is a globally prevalent public health issue [1]. Children who experience childhood maltreatment (defined as any form of sexual, physical, or emotional abuse and neglect), are more likely to have adverse physical and mental health outcomes including anxiety, depression, and substance use problems during adolescence [2–4]. Approximately one in four children and adolescents reported experiencing maltreatment in their lifetime in a national survey in the United States [1]. There is growing evidence that there is a high prevalence of childhood maltreatment in China [5]. A recent meta-analysis reported that 30% of elementary and middle school students in China have experienced emotional abuse, 12% sexual abuse, 20% physical abuse, 44% emotional neglect, and 47% physical neglect [6]. The rates of childhood maltreatment are disproportionately higher among youth from rural or low-income communities and migrant families [7].

Childhood maltreatment affects the development of nervous, endocrine, and immune systems, resulting in impaired cognitive, social, and emotional functioning [4, 8, 9]. As maltreated youth navigate through a progression of developmental tasks during adolescence, they are more likely to exhibit health-damaging behaviors such as tobacco and alcohol use [8, 10]. There is evidence that childhood maltreatment is associated with early initiation of alcohol use [11, 12], more frequent alcohol and tobacco use [13, 14], and a higher likelihood of binge drinking during adolescence and young adults [15].

The ecological systems theory emphasizes that youth development is influenced by multiple layers of environments including schools and neighborhoods—two contexts where youth spend much of their time [16]. Based on this theory, we expect that the developmental pathways from childhood maltreatment to adolescent substance use are moderated by experiences in neighborhoods and schools [16]. Perceptions of connection, which refer to youths’ feelings of sharing strong social ties, cohesion, and social support with their schools and neighborhoods, play important roles on youth mental and physical wellbeing and substance use behaviors [17]. Previous evidence suggests that feeling poorly connected in the neighborhood was associated with heightened levels of externalizing behaviors among youth with a history of childhood maltreatment, but not among non-maltreated youth [18]. Yet, according to the differential susceptibility theory, youth who are more negatively affected by an adverse environment might also benefit more from a positive environment [19]. It is possible that youth with a history of childhood maltreatment are also more susceptible to positive social contexts (e.g., a stronger sense of connection to the school and neighborhood contexts may be particularly beneficial in lowering risk behaviors among maltreated youth), although this proposition remains less examined in adolescent substance use research. As perceived neighborhood and school connection are modifiable factors, understanding their protective effects on substance use behaviors can help inform intervention programs designed to mitigate or counteract the negative impact of childhood maltreatment on subsequent substance use and health risks.

## The current study

We had three major goals in this longitudinal study. The first goal was to examine the unique effects of multiple forms of childhood maltreatment on adolescent tobacco and alcohol use. Including these multiple maltreatment forms (neglect and emotional, physical, and sexual abuse) afforded us the opportunity to assess if some forms of abuse are more strongly associated with subsequent substance use than are others. Second, despite the high prevalence of childhood maltreatment in Chinese children and adolescents, little is known about the impact of childhood maltreatment on tobacco and alcohol use for Chinese youth, particularly those who grew up in low-income rural communities (20, 21). This is the first study to evaluate how childhood maltreatment experiences are associated with developmental trajectories of alcohol and tobacco use among Chinese adolescents. To examine the development of substance use, we assessed two primary outcomes (any drinking and smoking in the past month) and a secondary outcome (binge drinking in the past month) every six months over one year. Third, we explored the possibility that perceived neighborhood or school connection moderated the association between maltreatment and later tobacco and alcohol use. We expected that positive school and neighborhood environments could protect maltreated youth against risk of substance use [16, 18, 19]. The current study fills these gaps by analyzing childhood maltreatment and longitudinal substance use data from a large sample of adolescents from four elementary and middle schools in China. In addition to addressing the three major questions of this study, we also explored the possibility that sex and grade moderated the associations between maltreatment and substance use.

## Methods

### Participants

Adolescents participating in this study came from an ongoing longitudinal study examining the effects of early life adversity on adolescent development conducted in a low-income rural county in Eastern China’s Anhui province. The gross regional product per capita of $2,526 in 2019 was one fifth of the gross domestic product per capita in China that year. The unemployment rate is high, and as a result, many adults migrate to cities leaving their children in their hometown. The research team contacted the school administrators and head teachers of two elementary and two middle schools for their permission for recruitment. All students in Grade 4 and 5 of the elementary schools, and Grade 7 and 8 of the middle schools, were invited to participate in the study with no criteria for exclusion. Written informed child assent and parental consent were collected from 3,079 adolescents aged 9 to 13 years at baseline with 100% consent rate.

After the initial assessment in December 2019 (T1), longitudinal follow-up questionnaires were collected approximately 6 to 7 months later in June 2020 (T2), and then again approximately 1 year after baseline in November and December 2020 (T3). This study included participants who had at least one follow-up assessment (*n* = 223 lost to attrition). We further excluded those missing substance use data (*n* = 46) or missing a covariate (*n* = 220), resulting in a final analytic sample of 2,594 participants (see Additional file 1: Figure S1 for a flow chart of study sample inclusion). Compared to the analyzed sample, participants excluded from analysis were more likely to report past-month drinking (*χ*^2^ [1] = 14.6, *p* < 0.001), binge drinking (*χ*^2^ [1] = 23.6, *p* < 0.001), and smoking (*χ*^2^ [1] = 33.7, *p* < 0.001); the two groups did not differ in self-reported abuse or neglect or demographic characteristics such as sex, grade, and parental living status.

At each wave of assessment, the participants were group-administered self-report questionnaires assessing demographic characteristics, maltreatment history, psychosocial functioning, and health behaviors in a classroom. Psychology faculty members and graduate students administered the questionnaires. During the collection of data, explanations were provided to participants to ensure that they understood the measures and procedures. The study was approved by the research ethics committee of Beijing Normal University (IRB #2019111270072).

### Measures

#### Past-month alcohol and tobacco use

At each of the three assessments, participants reported their past-month alcohol and tobacco use: “On how many days did you drink alcohol?”; and “On how many days did you smoke cigarettes?”. Binge drinking in the past month was assessed by one question: “On how many days did you drink five or more alcoholic drinks in a row?”, which was consistent with prior literature on binge drinking in this age group [22]. Response options were: 0 days; 1–2 days; 3–5 days; 6–9 days; 10–19 days; 20–29 days; and 30 days. These three variables were dichotomized to index whether the participant drank, binge drank, or smoked on any days during the past month (1 = yes; 0 = no). We used this threshold given the relatively low frequency of alcohol and cigarette use in our sample (for example, 89.4% of participants drank alcohol on 0 days, with 8.5% drank on 1 to 5 days and another 2.1% on 6 days or more).

#### Childhood maltreatment

Childhood Trauma Questionnaire (CTQ), a widely used 28-item self-report questionnaire, was used at baseline to assess the history of childhood maltreatment in participants [23]. The reliability and validity of the CTQ have been supported extensively for use in retrospective studies as childhood abuse and neglect [23–25]; The Chinese version of the CTQ has been validated in Chinese adolescent samples [24]. Participants were asked to respond to five statements for each type of abuse and neglect. Participants rated the frequency that they experienced each event when they “were growing up” on a 5-point scale ranging from 1 (never true) to 5 (very often true). The internal consistency was acceptable for sexual, physical, and emotional abuse and emotional neglect (Cronbach’s alpha ranged from 0.73 to 0.85). The internal consistency was 0.45 for physical neglect, which was similar to that obtained in studies in China, the U.S. and Sweden [24–26]. To address this, the physical and emotional neglect scales were combined to form a general neglect scale (α = 0.79), a procedure also used by others [27].

#### Perceived school and neighborhood connection

Perceived school and neighborhood connection were measured at baseline with the Positive Youth Development Scale Chinese Version [28]. This 51-item scale assessing positive development attributes was developed and validated in Chinese adolescents [28]. All items were rated on a five-point scale, ranging from 1 (never true) to 5 (almost always true). The school connection subscale included 6 items (e.g., “I get a lot of encouragement in school”, “I am proud of my school”). The neighborhood connection subscale was assessed by 3 items (e.g., “My neighborhoods care about me”, “My neighbors think they can trust me”). Responses were averaged to create school connection (α = 0.82) and neighborhood connection (α = 0.77) scores.

#### Covariates

Covariates included sex, grade, parental living status, and subjective socioeconomic status (SES). Sex was coded as 1 (male) and 0 (female). Grade was included as a dichotomous variable of 1 (middle school; Grade 7 or 8) and 0 (elementary school; Grade 4 or 5). Adolescents were asked whether their parents left their rural hometown and moved to cities for work over the past six consecutive months; this was coded as mutually exclusive categories: 2 = both parents absent; 1 = one parent absent; and 0 = none. Subjective SES was a continuous measure of adolescents’ perception of their families’ position in the social hierarchy; scores on this scale ranged from 1 to 10 [29].

### Statistical analysis

Multilevel growth curve modeling with a logit link function was used to model repeated measures of past-month drinking, binge drinking, and smoking over three time points [30]. Multilevel growth curve modeling is appropriate given the hierarchical structure of the data such that multiple assessments over time (Level 1) were nested within persons (Level 2). The wave of assessment, centered at T1, was used as the time metric (coded as T1 = 0, T2 = 1, and T3 = 2).

In Model 1, we examined the effect of childhood maltreatment on adolescent alcohol and tobacco use at baseline (random intercept) as well as its change over the study period (random slope). The fixed effects of emotional, physical, and sexual abuse and neglect as predictors of the random intercept afforded the ability to test whether abuse or neglect was independently associated with risk of alcohol and tobacco use at baseline. The random slope of risk behaviors allowed us to test the extent to which abuse or neglect was associated with an increased growth rate of alcohol and tobacco use. We adjusted for a set of personal, family, and contextual covariates, including sex, grade, parental living status, subjective SES, school connection, and neighborhood connection. In Model 2, the interaction between perceived school connection with childhood maltreatment variables to Model 1 was added. In Model 3, the interaction between perceived neighborhood connection with childhood maltreatment variables was added. The two primary outcomes (i.e., any drinking and smoking in the past month) were examined in main analyses, and binge drinking in the past month was included in the secondary analysis. In supplementary analyses, the possibility that the association between childhood maltreatment and alcohol and tobacco use was moderated by sex and grade was explored.

Consistent with recommendations, all models were estimated using restricted maximum likelihood and an unstructured covariance matrix [31]. All participants were included in the estimation of model parameters, as this approach can handle any participant with outcome data on at least one occasion. Continuous variables were standardized before analysis to facilitate interpretation. Analyses were conducted in Stata 15.0 [32]. A two-sided *p* < 0.05 was considered statistically significant.

## Results

Table 1 describes participant characteristics at baseline. Participants were between 9 and 13 years at baseline (*M* = 12.46, *SD* = 1.70 years); 55.8% were boys, and 62.3% were from middle school. Participants reported an average score of 7.85 (*SD* = 3.40) on emotional abuse, 6.68 (*SD* = 3.04) on physical abuse, 6.40 (*SD* = 2.94) on sexual abuse, and 10.09 (*SD* = 3.70) on neglect. Severity classification using established CTQ scale cut-points for each maltreatment type is provided in Table 2. The prevalence of moderate or severe maltreatment was 10.1% for emotional abuse, 12.2% for physical abuse, 17.9% for sexual abuse, and 45.8% for neglect. The participants reported relatively low prevalence of past-month drinking (T1 10.2%; T2 11.4%; T3 10.5%), binge drinking (T1 5.5%; T2 4.8%; T3 5.3%), and smoking (T1 4.9%; T2 3.1%; T3 4.1%; see Additiona file 1: Table S1). Additiona file 1: Table S2 presents Pearson’s correlations between study variables. All forms of abuse and neglect were positively correlated with adolescent alcohol and tobacco use at baseline, with small to moderate correlations.

**Table 1:** Participant characteristics at baseline (N = 2594)

Variables	M (SD)	Frequency (%)
Age in years	12.46 (1.70)	-
Gender		
Female	–	1146 (44.2)
Male	–	1448 (55.8)
Grade		
4th grade	–	435 (16.7)
5th grade	–	543 (20.9)
7th grade	–	808 (31.2)
8th grade	–	808 (31.2)
Parental absence		
Both parents absent	–	1217 (46.9)
One parent absent	–	640 (24.7)
None	–	737 (28.4)
Subjective SES	5.47 (1.59)	–
Perceived school connection	3.53 (0.86)	–
Perceived neighborhood connection	3.49 (1.10)	–
Childhood maltreatment subscales		
Emotional abuse	7.85 (3.40)	–
Physical abuse	6.68 (3.04)	–
Sexual abuse	6.40 (2.94)	–
Neglect	10.09 (3.70)	–
Drinking in the past month		
0 days	–	2329 (89.8)
1–5 days	–	211 (8.1)
6 days or more	–	54 (2.1)
Binge drinking in the past month		
0 days	–	2452 (94.5)
1–5 days	–	122 (4.7)
6 days or more	–	20 (0.8)
Smoking in the past month		
0 days	–	2466 (95.1)
1–5 days	–	88 (3.4)
6 days or more	–	40 (1.5)

Mean and standard deviation are shown for continuous variables; frequency and percentages are shown for categorical variables. Drinking, binge drinking and smoking variables were dichotomized in subsequent analyses to indicate any use in the past month

*SES* socioeconomic status

**Table 2:** Descriptive statistics for reports of childhood maltreatment

CTQ subscales	CTQ continuous scores		Classification according to severity *n*(%)			
	Min-Max	M (SD)	None to minimal		Moderate	Severe
Emotional abuse	5–25	7.85 (3.40)	2333 (89.94)		159 (6.13)	102 (3.93)
Physical abuse	5–25	6.68 (3.04)	2277 (87.78)		151 (5.82)	166 (6.40)
Sexual abuse	5–25	6.40 (2.94)	2131 (81.15)		310 (11.95)	153 (5.90)
Neglect	5–25	10.09 (3.70)	1405 (54.16)		679 (26.18)	510 (19.66)

Neglect continuous score was the average of physical and emotional neglect. Cut points for none-to-minimal, moderate, and severe were applied to facilitate interpretation according to the Childhood Trauma Questionnaire (CTQ) scoring manual (Bernstein et al., 2003): emotional abuse (≤ 12, 13–15, ≥ 16); physical abuse (≤ 9, 10–12, ≥ 13); sexual abuse (≤ 7, 8–12, ≥ 13); emotional neglect (≤ 14, 15–17, ≥ 18); physical neglect (≤ 9, 10–12, ≥ 13). The categorization of severe (or moderate) neglect was based on whether individuals met the designated cutoff scores on either the physical or emotional subscale

**Fig. 1:**
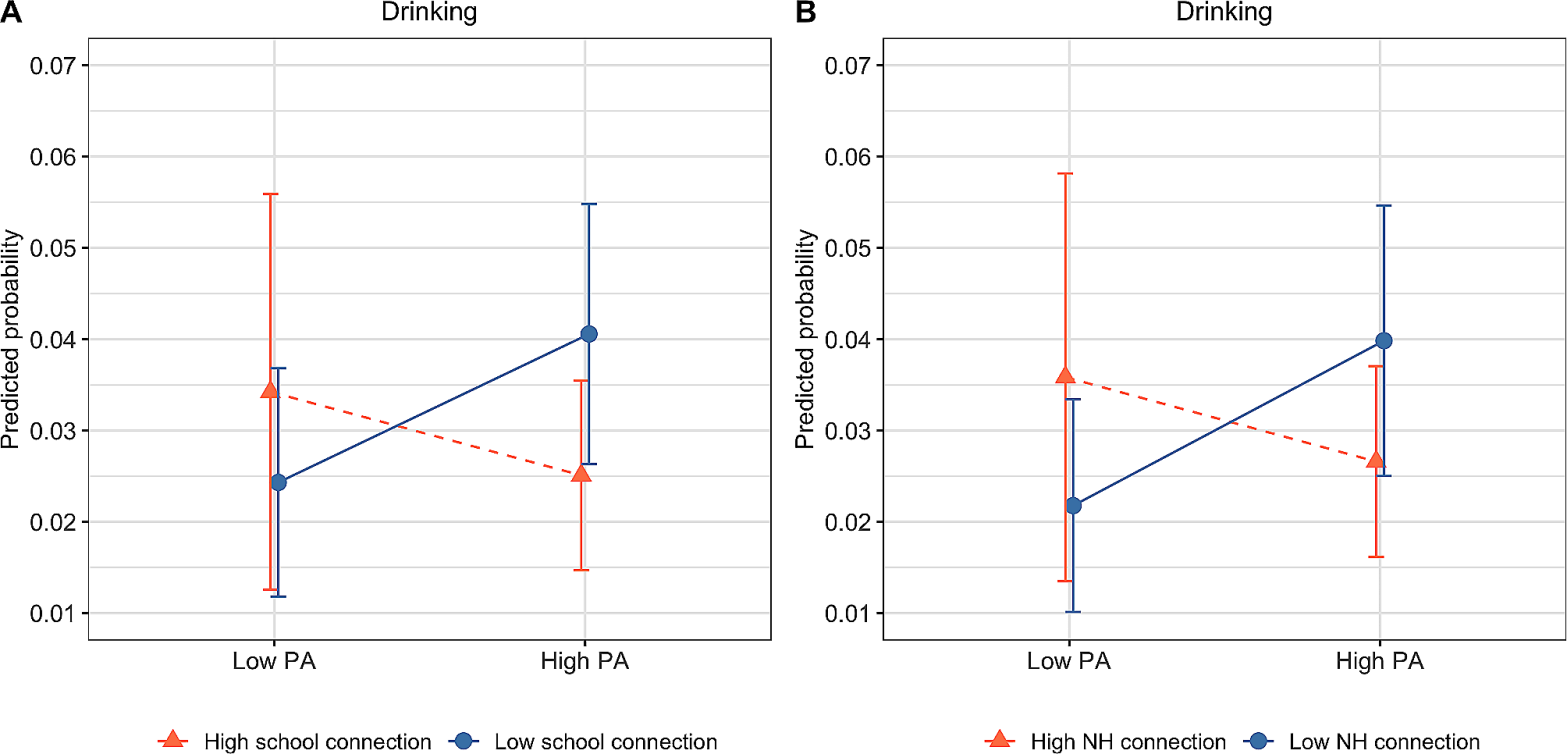
Interaction effects between perceived school connection (left) and neighborhood connection (right) with physical abuse (PA) in predicting adolescent past-month drinking behavior. *NH *neighborhood

Our main analyses (Table 3) revealed that both sexual abuse and neglect were respectively associated with increased odds of alcohol use at baseline (OR = 1.53, 95% CI 1.19–2.03, *p* < 0.001; OR = 1.52, 95% CI 1.06–1.90, *p* < 0.05, Model 1). Emotional abuse and physical abuse were not uniquely associated with past-month drinking. Although past-month drinking increased over the study period (OR = 1.17, 95% CI 1.03–1.33, *p* < 0.05), none of the four maltreatment forms was associated with the rate of increase. There was a significant interaction between physical abuse and perceived school connection in predicting drinking (interaction *p* < 0.05, Additional file 1: Table S2). As shown in Fig. 1a, physical abuse was associated with a higher risk of alcohol use only among youth who perceived low school connection, but not among those who perceived high school connection. There was a similar interaction effect between perceived neighborhood connection and physical abuse (interaction *p* < 0.05**)**, such that physical abuse was associated with a higher risk of drinking only among youth who perceived low neighborhood connection, but not among those who perceived high neighborhood connection (Fig. 1b**)**.

**Table 3:** Multilevel growth curve models of the association between childhood maltreatment and trajectories of past-month substance use from T1 to T3 (n = 2594)

	Drinking	Smoking
Predictors	OR (95% CI)	OR (95% CI)
Time	1.17 (1.03–1.33) *	1.06 (0.87–1.30)
Emotional abuse (EA)	1.27 (0.90–1.67)	0.96 (0.63–1.46)
Physical abuse (PA)	1.23 (0.91–1.68)	1.59 (1.08–2.34) *
Sexual abuse (SA)	1.53 (1.19–2.03) ***	1.82 (1.30–2.55) ***
Neglect	1.52 (1.06–1.90) *	2.02 (1.34–3.02) ***
Abuse/neglect x time		
EA x time	0.92 (0.77–1.11)	0.99 (0.76–1.29)
PA x time	0.92 (0.77–1.10)	0.87 (0.69–1.10)
SA x time	0.88 (0.77–1.05)	0.95 (0.77–1.16)
Neglect x time	0.86 (0.73–1.01)	0.79 (0.62–1.00)
Covariates		
Male (ref. = female)	2.31 (1.68–3.31) ***	5.74 (3.32–9.91) ***
Middle school (ref. = primary school)	4.90 (3.60–7.64) ***	6.30 (2.64–14.98) ***
Parental absence (ref. = none)		
One	0.61 (0.39–0.97) *	0.81 (0.44–1.50)
Both	0.99 (0.66–1.39)	0.82 (0.49–1.39)
Subjective SES	1.05 (0.89–1.24)	1.26 (1.01–1.58)*
School connection	0.87 (0.72–1.04)	0.84 (0.65–1.09)
Neighborhood connection	0.94 (0.80–1.15)	0.90 (0.70–1.15)

*OR *odds ratios; *95% CI*  95% confidence intervals; *SES *socioeconomic status.

*** *p* < 0.001; ** *p* < 0.01; * *p* < 0.05

For past-month smoking (Table 3), physical abuse, sexual abuse, and neglect were each associated with increased odds of smoking at baseline (OR = 1.59, 95% CI 1.08–2.34, *p* < 0.05; OR = 1.82, 95% CI 1.30–2.55, *p* < 0.001; and OR = 2.02, 95% CI 1.34–3.02, *p* < 0.001, respectively). There was no significant increase in the risk of smoking over time, and the four forms of maltreatment were not associated with the rate of change. Perceived school or neighborhood connection did not moderate these associations.

Results of our secondary analysis on past-month binge drinking were similar to our main model of past-month smoking (Additional file 1: Table S3). Physical abuse, sexual abuse, and neglect were each associated with increased odds of binge drinking at baseline (OR = 1.64, 95% CI 1.17–2.37, *p* < 0.01; OR = 1.85, 95% CI 1.40–2.61, *p* < 0.001; and OR = 1.48, 95% CI 1.06–2.18, *p* < 0.05, respectively). The risk of binge drinking did not significantly increase over time and none of the four forms of maltreatment were associated with the rate of change. These associations were not moderated by perceived school or neighborhood connection.

Supplementary analyses examined the possibility that the associations between child maltreatment and alcohol and tobacco use were moderated by sex and grade (Supplementary eTables 4 and 5). There was a significant interaction effect between physical abuse and sex in predicting past-month drinking at baseline (interaction *p* < 0.05). Specifically, physical abuse was more strongly associated with boys’ than with girls’ drinking (Additional file 1: Fig. S2). In addition, there was a significant interaction effect between sexual abuse and grade in predicting past-month binge drinking at baseline (interaction *p* < 0.05). Sexual abuse was more strongly associated with binge drinking among middle school than elementary school youth.

## Discussion

This study examined how childhood maltreatment is associated with the longitudinal trajectories of tobacco and alcohol use in a large sample of low-income, rural Chinese adolescents. The results yielded two novel contributions. First, there are unique effects of sexual abuse and neglect on subsequent risks of alcohol and tobacco use, independent of personal, family, and contextual characteristics. Second, the positive aspects of the school and neighborhood environment help to buffer the risks of alcohol use associated with childhood physical abuse experiences. This finding has implications for designing and implementing targeted school and neighborhood-based substance use preventions and interventions.

Different forms of childhood maltreatment were independently associated with baseline adolescent substance use. Sexual abuse and neglect were more strongly associated with substance use than was physical or emotional abuse. The finding that childhood sexual abuse was associated with increased risk of past-month drinking, binge drinking, and smoking is consistent with prior research documenting the negative consequences of sexual abuse [4]. Childhood sexual abuse can impair stress response systems, leading to maladaptive coping behaviors, poor decision-making and low self-regulation [9]. Victims of sexual abuse may be more likely to use alcohol or tobacco as a coping mechanism [33, 34]. We also found sexual abuse was more strongly associated with smoking among youth in middle school than those in elementary school. While the effects of sexual abuse on some aspects of adjustment (e.g., internalizing problems) manifest early, its effect on other domains such as substance use may emerge later in adolescence, possibly through a cascade of developmental challenges involving sexual risk behaviors, delinquent behaviors, and involvement with deviant peers [4].

Neglect uniquely predicted adolescent past-month drinking, binge drinking, and smoking, above and beyond the effects of physical, sexual, and emotional abuse. The effect size of neglect was similar to that of sexual abuse for drinking, and even stronger for smoking. This finding is consistent with past research that the negative effect of neglect on adolescent health and well-being was similar to or even greater than abuse [14, 35, 36]. Neglect is a severe form of early deprivation and may lead to substance use via insecure attachment, which has been associated with emotional dysregulation, externalizing behaviors, and psychosocial adjustment difficulties [37]. Neglect was the most common form of maltreatment in this sample, a result consistent with other research [38]. The high rate of neglect in our sample may reflect the fact that many parents migrated to cities, leaving their children to a single parent, a grandparent, or another adult. Considering that recent research has found distinct components of neglect (e.g., not providing emotional support and affect, exposing youth to risky situations, or inadequate monitoring) that independently influence the likelihood to exhibit health-damaging behaviors [36], it is important for future research to examine the specific component of neglect that influences the well-being and development of children and adolescents.

In prior studies it has also been found that adolescents who had experienced emotional abuse were more likely to initiate alcohol and tobacco early and to use these substances more frequently [12, 33]. Surprisingly, we found emotional abuse was not significantly associated with drinking, binge drinking, or smoking. Physical abuse predicted binge drinking and smoking; however, it was only associated with drinking for boys, but not for girls. Previous findings are mixed regarding sex differences in the effects of physical abuse [39, 40]. Future research is needed to explore the effects of abuse with consideration of multiple factors including cultural values and norms, the developmental stage of participants, and features of physical and emotional abuse such as severity and chronicity.

Consistent with the ecological systems theory, school and neighborhood connections moderated the developmental pathways from early physical abuse to adolescent drinking behavior [16]. Although it was previously found that individuals with a history of childhood maltreatment have heightened vulnerability to adverse environment influences [41], there has been less empirical support for the protective effects of positive environments. For adolescents who experienced childhood physical abuse, feelings of connection to peers and adults in school and neighborhood were associated with a reduced risk for alcohol drinking in adolescence. One plausible explanation is physical abuse is more common for children who exhibit a difficult temperament or other personality or biological traits. As posited by the differential susceptibility theory, these traits may confer a heightened susceptibility to environmental influences, for better and for worse [42]. It is also likely that early physical abuse alters the physiological systems involved in stress responsivity, subsequently enhancing youth sensitivity to their environments [19]. It will be valuable for future research to further examine possible mechanisms of environmental susceptibility among youth who experienced physical abuse. Nonetheless, our findings underscore the important role of a positive context in cultivating resilience among physically abused youth. Policymakers and school administrators may consider interventions geared towards improving overall school and neighborhood connectedness given its potential to effectively mitigate the negative developmental sequalae affecting youth who experienced early-life physical abuse. It is also important to note that the moderating effects of school and neighborhood connections appear limited to physical abuse; thus, caution is needed in interpreting these findings. The potential interaction effects between abuse or neglect experiences and school and neighborhood contexts in predicting adolescent substance use and other developmental outcomes warrant further exploration.

The prevalence rates of 10.2% for past-month drinking and 4.9% for past-month smoking are similar to those found in prior studies of Chinese adolescents [43, 44]. For example, a nationally representative survey of Chinese adolescents yielded rates of past-month smoking of 3.9% for middle school students [43]. In another national survey to middle and high school students in eight Chinese provinces, 11.9% of adolescents reported using alcohol in the past month [44]. We also found that rates of alcohol and tobacco use were higher in middle school than in primary school and higher among boys than girls, patterns consistent with results from other studies [43, 44]. We observed that the proportion of youth in our sample who endorsed past-month alcohol use increased, while it remained unchanged for tobacco use, suggesting that exposures to alcohol and tobacco lasted or even increased over the study period. None of childhood maltreatment forms predicted the rate of change of drinking or smoking behaviors, suggesting that sexual and physical abuse and neglect are associated with higher alcohol and tobacco use at baseline, but this effect may stabilize over time. There are two plausible explanations for the lack of association for substance use change. First, participants who are lost to attribution have higher baseline rates of substance use; thus, our analysis does not capture high-risk behaviors that potentially persist or increase over time and likely underestimates the true impact of childhood maltreatment on the substance use trajectory. Second, there may exist distinct trajectory patterns of substance use within maltreated youth (e.g., some youths have increasing or persistent use, whereas others have decreasing use). Further research to explore developmental patterns and/or risk profiles of substance use (e.g., lifetime exposure, exposure amount, cumulative amount, age of initiation, duration of use) among maltreated samples is warranted.

### Limitations and implications for future research

There are several limitations of this research that suggest the need for future research. First, the study relied on self-reported measurement of the key variables. For example, childhood maltreatment was retrospectively reported by youth, which may be subject to recall bias (e.g., current life situations could influence the recall of past experiences) and could be underestimated given social stigma and victim-blaming faced by victims [45]. Future research may consider using multiple data sources including official records, self-and parent-reported maltreatment data, and parental, teacher, and peer perceptions of youth substance use. Also, the self-report measure of childhood maltreatment did not allow an examination of the age of onset, perpetrator, and chronicity of the maltreatment exposure, which should be considered in future research on childhood maltreatment. Given that abuse and neglect often co-occur, it would be useful to examine the complexity of maltreatment experiences using person-centered analysis such as the latent profile analysis and evaluate how complex patterns of maltreatment experiences relate to adolescent substance use trajectories.

Second, although the validity of the CTQ scale has been supported in prior research [23, 24], the internal consistency of the physical neglect subscale was only moderate. The lower internal consistency of this subscale was not specific to our study but rather common among community samples of adolescents across countries [24–26]. We therefore used a combined neglect measure because it provided an acceptable internal consistency and was consistent with the broad conceptualization of neglect used in previous research [27, 35, 46]. In future research it would be helpful to distinguish physical and emotional forms of neglect, using case records, diagnostic interview measures, or other validated multidimensional measures of neglect.

Third, this study assessed alcohol and tobacco trajectory over the period of one year, which may not fully capture the lasting effect of childhood maltreatment. Although we found abuse or neglect did not predict increases in the use of these substances, it is possible that the effects of early maltreatment experiences extend beyond a one-year period and persist into late adolescence and adulthood [47]. Future studies with more years of follow-up data are needed to evaluate the long-term effects of childhood maltreatment on substance use behaviors.

Fourth, given that the protective effects of school and neighborhood connection were only observed in the link between physical abuse and alcohol use, it is important to interpret the school and neighborhood effect with caution. We encourage future research to assess if context matters via various school and neighborhood processes (i.e., perceived safety, access to resources, built environment) and across different developmental outcomes and samples from diverse backgrounds among maltreated youth.

Finally, this study was conducted in a low-income rural area of Eastern China and the generalizability of the reported findings to other populations in China and other regions of the world should be interpreted cautiously. For example, there are considerable differences in economic development and societal norms pertaining to tobacco and alcohol use across China. Results from a recent meta-analysis showed that tobacco use is more prevalent in Eastern China than is in either Central China or Western China [48]. Future research should replicate current findings in different parts of China and in other societies. Nonetheless, our use of a low-income rural Chinese youth sample contributes to the understanding of the impact of childhood maltreatment in underserved populations.

## Conclusions

This study of a large, understudied sample of adolescents in low-income rural China, demonstrates how childhood sexual abuse, physical abuse, and neglect are each prospectively associated with risks of adolescent alcohol and tobacco use. Our findings also highlight the protective roles of the neighborhood and school connections in mitigating the negative effect of physical abuse on adolescent alcohol use. This research contributes to the literature of adolescent development by examining unique effects of multiple forms of childhood maltreatment in Chinese rural settings. Our findings underscore the need for more comprehensive tools to identify maltreatment exposures and their effects in low-income rural communities. Given that neighborhood and school connection are modifiable factors, policymakers may design and implement effective intervention programs that increase feelings of trust, closeness, and belongingness among physically abused youth. More work needs to address crucial social forces (i.e., poverty, lack of access to resources) that lead to health inequalities and interpersonal violence among children and adolescents.

### Electronic supplementary material

**Additional file 1: Figure S1. **Inclusion of the study participants for analysis in a study of the association between childhood maltreatment and substance use trajectories in adolescents. **Figure S2**. Interaction effect between sex and physical abuse (PA) predicting past-month drinking (left) and interaction effect between grade and sexual abuse (SA) in predicting past-month binge drinking (right). **Table S1. **Frequencies of past-month drinking, smoking, and binge drinking from T1 to T3. **Table S2.** Correlation between study variables at baseline.  **Table S3. **Multilevel growth curve models of the association between childhood maltreatment and trajectories of past-month binge drinking from T1 to T3 (n=2594). **Table S4.** Moderating effects of school and neighborhood connection in the association between childhood maltreatment and past-month drinking, binge drinking and smoking. **Table S5.** Moderating effects of sex and grade in the association between childhood maltreatment and past-month drinking, binge drinking and smoking. **Figure S2**. Interaction effect between sex and physical abuse (PA) predicting past-month drinking (left) and interaction effect between grade and sexual abuse (SA) in predicting past-month binge drinking (right). 

## Data Availability

The data that support the findings of this study are available from the corresponding author upon reasonable request.

## Abbreviations

CI: Confidence intervals
OR: Odds ratios
CTQ: Child trauma questionnaire
SES: Socioeconomic status
SD: Standard deviation

## References

[1] Finkelhor D, Turner HA, Shattuck A, Hamby SL (2015). Prevalence of childhood exposure to violence, crime, and abuse: results from the national survey of children’s exposure to violence. JAMA Pediatr.

[2] Humphreys KL, LeMoult J, Wear JG, Piersiak HA, Lee A, Gotlib IH (2020). Child maltreatment and depression: a meta-analysis of studies using the childhood trauma questionnaire. Child Abuse Negl.

[3] Sokol RL, Gottfredson NC, Poti JM, Shanahan ME, Halpern CT, Fisher EB (2019). Sensitive periods for the Association between childhood maltreatment and BMI. Am J Prev Med.

[4] Negriff S, Gordis EB, Susman EJ, Kim K, Peckins MK, Schneiderman JU (2020). The young adolescent project: a longitudinal study of the effects of maltreatment on adolescent development. Dev Psychopathol.

[5] Fang X, Fry DA, Ji K, Finkelhor D, Chen J, Lannen P (2015). The burden of child maltreatment in China: a systematic review. Bull World Health Organ.

[6] Wang L, Cheng H, Qu Y, Zhang Y, Cui Q, Zou H (2020). The prevalence of child maltreatment among Chinese primary and middle school students: a systematic review and meta-analysis. Soc Psychiatry Psychiatr Epidemiol.

[7] Gao Y, Atkinson-Sheppard S, Liu X (2017). Prevalence and risk factors of child maltreatment among migrant families in China. Child Abuse Negl.

[8] Cicchetti D, Handley ED (2019). Child maltreatment and the development of substance use and disorder. Neurobiol Stress.

[9] Lupien SJ, Maheu F, Tu M, Fiocco A, Schramek TE (2007). The effects of stress and stress hormones on human cognition: implications for the field of brain and cognition. Brain Cogn.

[10] Lansford JE, Dodge KA, Pettit GS, Bates JE (2010). Does physical abuse in early childhood predict substance use in adolescence and early adulthood?. Child Maltreat.

[11] Shin SH, Jiskrova GK, Yoon SH, Kobulsky JM (2020). Childhood maltreatment, motives to drink and alcohol-related problems in young adulthood. Child Abuse Negl.

[12] Hamburger ME, Leeb RT, Swahn MH (2008). Childhood maltreatment and early alcohol use among high-risk adolescents. J Stud Alcohol Drug.

[13] Tonmyr L, Thornton T, Draca J, Wekerle C (2010). A review of childhood maltreatment and adolescent substance use relationship. Curr Psychiatry Reviews.

[14] Diaz A, Shankar V, Nucci-Sack A, Linares LO, Salandy A, Strickler HD (2020). Effect of child abuse and neglect on risk behaviors in inner-city minority female adolescents and young adults. Child Abuse Negl.

[15] Bayly BL, Hung YW, Cooper DK (2022). Age-varying associations between child maltreatment, depressive symptoms, and frequent heavy episodic drinking. J Youth Adolesc.

[16] Bronfenbrenner U, Friedman SL, Wachs TD (1999). Environments in developmental perspective: Theoretical and operational models. Measuring environment across the life span: Emerging methods and concepts.

[17] Wilkinson A, Lantos H, McDaniel T, Winslow H (2019). Disrupting the link between maltreatment and delinquency: how school, family, and community factors can be protective. BMC Public Health.

[18] Yonas MA, Lewis T, Hussey JM, Thompson R, Newton R, English D (2010). Perceptions of neighborhood collective efficacy moderate the impact of maltreatment on aggression. Child Maltreat.

[19] Belsky J, Pluess M (2009). Beyond diathesis stress: Differential susceptibility to environmental influences. Psychol Bull.

[20] Chen J, Dunne MP, Han P (2006). Child sexual abuse in Henan Province, China: associations with sadness, suicidality, and risk behaviors among adolescent girls. J Adolesc Health.

[21] Lin D, Li X, Fan X, Fang X (2011). Child sexual abuse and its relationship with health risk behaviors among rural children and adolescents in Hunan. China Child Abuse Negl.

[22] Johnston LD, Miech RA, O’Malley PM, Bachman JG, Schulenberg JE, Patrick ME. Monitoring the future national survey results on drug use, 1975–2021: Overview, key findings on adolescent drug use.

[23] Bernstein DP, Stein JA, Newcomb MD, Walker E, Pogge D, Ahluvalia T (2003). Development and validation of a brief screening version of the childhood trauma Questionnaire. Child Abuse Negl.

[24] He J, Zhong X, Gao Y, Xiong G, Yao S (2019). Psychometric properties of the Chinese version of the childhood trauma questionnaire-short form (CTQ-SF) among undergraduates and depressive patients. Child Abuse Negl.

[25] Hagborg JM, Kalin T, Gerdner A (2022). The Childhood Trauma Questionnaire—Short Form (CTQ-SF) used with adolescents–methodological report from clinical and community samples. J Child Adolesc Trauma.

[26] Gerdner A, Allgulander C (2009). Psychometric properties of the Swedish version of the childhood trauma Questionnaire—Short form (CTQ-SF). Nord J Psychiatry.

[27] Scher CD, Stein MB, Asmundson GJ, McCreary DR, Forde DR (2001). The childhood trauma questionnaire in a community sample: psychometric properties and normative data. J Trauma Stress.

[28] Chai X, Wang J, Li X, Liu W, Zhao G, Lin D (2020). Development and validation of the Chinese positive youth development scale. Appl Dev Sci.

[29] Adler NE, Epel ES, Castellazzo G, Ickovics JR (2000). Relationship of subjective and objective social status with psychological and physiological functioning: preliminary data in healthy, white women. Health Psychol.

[30] Lee TK, Wickrama K, O’Neal CW (2018). Application of latent growth curve analysis with categorical responses in social behavioral research. Struct Equation Model Multidisciplinary J.

[31] Molenberghs G, Kenward M (2007). Missing data in clinical studies.

[32] StataCorp (2017). Stata Statistical Software: release 15. College Station.

[33] Negriff S (2018). Developmental pathways from maltreatment to risk behavior: sexual behavior as a catalyst. Dev Psychopathol.

[34] Jones DJ, Lewis T, Litrownik A, Thompson R, Proctor LJ, Isbell P (2013). Linking childhood sexual abuse and early adolescent risk behavior: the intervening role of internalizing and externalizing problems. J Abnorm Child Psychol.

[35] Duprey EB, Oshri A, Caughy MO (2017). Childhood neglect, internalizing symptoms and adolescent substance use: does the neighborhood context matter?. J Youth Adolesc.

[36] Kobulsky JM, Villodas M, Yoon D, Wildfeuer R, Steinberg L, Dubowitz H (2021). Adolescent neglect and health risk. Child Maltreat.

[37] Cyr C, Euser EM, Bakermans-Kranenburg MJ, Van IJzendoorn M (2020). Attachment security and disorganization in maltreating and high-risk families: a series of meta-analyses. Devenir.

[38] Fang X, Fry DA, Ji K, Finkelhor D, Chen J, Lannen P (2015). The burden of child maltreatment in China: a systematic review. Bull World Health Organ.

[39] Schilling EA, Aseltine RH, Gore S (2007). Adverse childhood experiences and mental health in young adults: a longitudinal survey. BMC Public Health.

[40] Jones DJ, Runyan DK, Lewis T, Litrownik AJ, Black MM, Wiley T (2010). Trajectories of childhood sexual abuse and early adolescent HIV/AIDS risk behaviors: the role of other maltreatment, witnessed violence, and child gender. J Clin Child Adolesc Psychol.

[41] Keyes KM, McLaughlin KA, Koenen KC, Goldmann E, Uddin M, Galea S (2012). Child maltreatment increases sensitivity to adverse social contexts: neighborhood physical disorder and incident binge drinking in Detroit. Drug Alcohol Depend.

[42] Belsky J, Pluess M (2009). Beyond diathesis stress: differential susceptibility to environmental influences. Psychol Bull.

[43] Zhao Y, Di X, Li S, Zeng X, Wang X, Nan Y (2022). Prevalence, frequency, intensity, and location of cigarette use among adolescents in China from 2013?14 to 2019: Findings from two repeated cross-sectional studies. Lancet Reg Health Western Pac.

[44] Wang J, Xie Y, Zhang Y, Xu H, Zhang X, Wan Y (2024). The relationship between cumulative ecological risk and health risk behaviors among Chinese adolescents. BMC Public Health.

[45] Tajima EA, Herrenkohl TI, Huang B, Whitney SD (2004). Measuring child maltreatment: a comparison of prospective parent reports and retrospective adolescent reports. Am J Orthopsychiatry.

[46] Kim K, Mennen FE, Trickett PK (2017). Patterns and correlates of co-occurrence among multiple types of child maltreatment. Child Family Social work.

[47] Raby KL, Roisman GI, Labella MH, Martin J, Fraley RC, Simpson JA (2019). The legacy of early abuse and neglect for social and academic competence from childhood to adulthood. Child Dev.

[48] Xiong PS, Xiong MJ, Liu ZX, Liu Y (2020). Prevalence of smoking among adolescents in China: an updated systematic review and meta-analysis. Public Health.

