# Supplementary material for: Childhood maltreatment and alcohol and tobacco use trajectories in rural Chinese adolescents
Source: Child Adolesc Psychiatry Ment Health. 2024 May 3;18:51. doi: 10.1186/s13034-024-00744-w (PMC11067222; doi:10.1186/s13034-024-00744-w)
Supplement: Supplementary file 1 — Additional file 1: Figure S1. Inclusion of the study participants for analysis in a study of the association between childhood maltreatment and substance use trajectories in adolescents. Figure S2. Interaction effect between sex and physical abuse (PA) predicting past-month drinking (left) and interaction effect between grade and sexual abuse (SA) in predicting past-month binge drinking (right). Table S1. Frequencies of past-month drinking, smoking, and binge drinking from T1 to T3. Table S2. Correlation between study variables at baseline. Table S3. Multilevel growth curve models of the association between childhood maltreatment and trajectories of past-month binge drinking from T1 to T3 (n=2594). Table S4. Moderating effects of school and neighborhood connection in the association between childhood maltreatment and past-month drinking, binge drinking and smoking. Table S5. Moderating effects of sex and grade in the association between childhood maltreatment and past-month drinking, binge drinking and smoking. Figure S2. Interaction effect between sex and physical abuse (PA) predicting past-month drinking (left) and interaction effect between grade and sexual abuse (SA) in predicting past-month binge drinking (right). [file 13034_2024_744_MOESM1_ESM.docx]

**Supplementary Materials**

**Table of Contents**

**Supplementary eFigure 1.** Inclusion of the study participants for analysis in a study of the association between childhood maltreatment and substance use trajectories in adolescents.

**Supplementary eTable 1.** Frequencies of past-month drinking, smoking, and binge drinking from T1 to T3.

**Supplementary eTable 2.** Correlation between study variables at baseline.

**Supplementary eTable 3.** Multilevel growth curve models of the association between childhood maltreatment and trajectories of past-month binge drinking from T1 to T3 (n=2,594).

**Supplementary eTable 4.** Moderating effects of school and neighborhood connection in the association between childhood maltreatment and past-month drinking, binge drinking and smoking.

**Supplementary eTable 5.** Moderating effects of sex and grade in the association between childhood maltreatment and past-month drinking, binge drinking and smoking.

**Supplementary eFigure 2**. Interaction effect between sex and physical abuse (PA) predicting past-month drinking (left) and interaction effect between grade and sexual abuse (SA) in predicting past-month binge drinking (right).

**Supplementary eFigure 1.** Inclusion of the study participants for analysis in a study of the association between childhood maltreatment and substance use trajectories in adolescents.

**Supplementary eTable 1.** Frequencies of past-month drinking, smoking, and binge drinking from T1 to T3 (n=2,594).

| Substance use in the past month | T1 | T2 | T3 |
| --- | --- | --- | --- |
| Any drinking |  |  |  |
| Yes | 10.2% | 11.4% | 10.5% |
| No | 89.8% | 88.6% | 89.5% |
| Any smoking |  |  |  |
| Yes | 4.9% | 3.1% | 4.1% |
| No | 95.1% | 96.9% | 95.9% |
| Any binge drinking |  |  |  |
| Yes | 5.5% | 4.8% | 5.3% |
| No | 94.5% | 95.2% | 94.7% |

**Supplementary eTable 2.** Correlation between study variables at baseline.

| Variables | 1 | 2 | 3 | 4 | 5 | 6 | 7 | 8 | 9 | 10 | 11 |
| --- | --- | --- | --- | --- | --- | --- | --- | --- | --- | --- | --- |
| 1. Emotional abuse | - |  |  |  |  |  |  |  |  |  |  |
| 2. Physical abuse | 0.63*** | - |  |  |  |  |  |  |  |  |  |
| 3. Sexual abuse | 0.51*** | 0.61*** | - |  |  |  |  |  |  |  |  |
| 4. Neglect | 0.38*** | 0.28*** | 0.21*** | - |  |  |  |  |  |  |  |
| 5. Drinking | 0.13*** | 0.12*** | 0.13*** | 0.10*** | - |  |  |  |  |  |  |
| 6. Binge drinking | 0.14*** | 0.18*** | 0.18*** | 0.10*** | 0.63*** | - |  |  |  |  |  |
| 7. Smoking | 0.13*** | 0.16*** | 0.16*** | 0.12*** | 0.45*** | 0.44*** | - |  |  |  |  |
| 8. Male | -0.02 | 0.13*** | 0.15*** | 0.05* | 0.07*** | 0.06** | 0.10*** | - |  |  |  |
| 9. Middle school | -0.03 | -0.18*** | -0.19*** | -0.09*** | 0.10*** | 0.06** | 0.08*** | -0.08*** | - |  |  |
| 10. Subjective SES | -0.10*** | -0.02 | 0.01 | -0.10*** | -0.03 | 0.01 | 0.01 | 0.01 | -0.17*** | - |  |
| 11. School connection | -0.18*** | -0.09*** | -0.06** | -0.32*** | -0.09*** | -0.09*** | -0.07** | -0.03 | -0.06** | 0.15*** | - |
| 12. Neighborhood connection | -0.15*** | -0.04* | -0.05** | -0.30*** | -0.04* | -0.04 | -0.03 | 0.03 | 0.04 | 0.11*** | 0.48*** |

Notes. *** *p*<0.001, ** *p*<0.01, ** *p*<0.05. SES = socioeconomic status.

**Supplementary eTable 3.** Multilevel growth curve models of the association between childhood maltreatment and trajectories of past-month binge drinking from T1 to T3 (n=2,594).

|  | Binge Drinking |
| --- | --- |
| Predictors | OR (95% CI) |
| Time | 1.19 (1.00 - 1.42) |
| Emotional abuse (EA) | 1.06 (0.69 - 1.45) |
| Physical abuse (PA) | 1.64 (1.17 - 2.37) ** |
| Sexual abuse (SA) | 1.85 (1.40 - 2.61) *** |
| Neglect | 1.48 (1.06 - 2.18) * |
| Abuse/neglect x Time |  |
| EA x Time | 0.91 (0.73 - 1.16) |
| PA x Time | 0.91 (0.73 - 1.11) |
| SA x Time | 0.91 (0.76 - 1.10) |
| Neglect x Time | 0.88 (0.69 - 1.06) |
| Covariates |  |
| Male (ref. = female) | 1.81 (1.21 - 2.75) ** |
| Middle school (ref. = primary school) | 3.34 (1.59 – 7.01) *** |
| Parental absence (ref. = none) |  |
| One | 0.61 (0.36 - 1.05) |
| Both | 0.60 (0.39 - 0.96) * |
| Subjective SES | 1.15 (0.94 – 1.40) |
| School connection | 0.83 (0.64 - 1.00) |
| Neighborhood connection | 0.87 (0.72 - 1.13) |

*Notes.* OR = odds ratios; 95% CI = 95% confidence intervals; SES = socioeconomic status. *** *p* < .001, ** *p* < .01, * *p* <.05.

**Supplementary eTable 4.** Moderating effects of school and neighborhood connection in the association between childhood maltreatment and past-month drinking, binge drinking and smoking.

|  | Drinking |  | Binge drinking |  | Smoking |  |
| --- | --- | --- | --- | --- | --- | --- |
| Predictors | Model 1 | Model 2 | Model 1 | Model 2 | Model 1 | Model 2 |
| Time | 1.17 (1.03 - 1.33) * | 1.17 (1.03 - 1.32) * | 1.19 (1.00 - 1.42) | 1.19 (1.00 - 1.42) | 1.05 (0.86 - 1.28) | 1.05 (0.86 - 1.28) |
| Emotional abuse (EA) | 1.32 (0.96 - 1.82) | 1.30 (0.95 - 1.79) | 1.12 (0.74 - 1.60) | 0.97 (0.66 - 1.43) | 0.95 (0.61 - 1.47) | 0.92 (0.60 - 1.42) |
| Physical abuse (PA) | 1.13 (0.82 - 1.55) | 1.17 (0.86 - 1.60) | 1.54 (1.07 - 2.21)* | 1.67 (1.16 - 2.38)** | 1.50 (1.01 - 2.24) * | 1.59 (1.08 - 2.34) * |
| Sexual abuse (SA) | 1.56 (1.19 - 2.05) ** | 1.56 (1.19 - 2.04)** | 1.87 (1.39 - 2.62)*** | 1.93 (1.42 - 2.64)*** | 1.92 (1.36 - 2.71) *** | 1.87 (1.33 - 2.62) *** |
| Neglect | 1.40 (1.06 - 1.90) * | 1.40 (1.04 - 1.88)* | 1.45 (1.03 - 2.15)* | 1.51 (1.05 - 2.18)* | 2.04 (1.36 - 3.07) *** | 2.02 (1.34 - 3.02) *** |
| Abuse/neglect x Time |  |  |  |  |  |  |
| EA x Time | 0.92 (0.77 - 1.10) | 0.92 (0.77 - 1.10) | 0.92 (0.73 - 1.15) | 0.92 (0.74 - 1.16) | 1.01 (0.78 - 1.31) | 1.01 (0.78 - 1.31) |
| PA x Time | 0.92 (0.77 - 1.10) | 0.92 (0.77 - 1.10) | 0.90 (0.73 - 1.11) | 0.90 (0.73 - 1.11) | 0.87 (0.69 - 1.10) | 0.87 (0.69 - 1.10) |
| SA x Time | 0.90 (0.77 - 1.05) | 0.89 (0.77 - 1.05) | 0.91 (0.76 - 1.10) | 0.91 (0.76 - 1.10) | 0.95 (0.77 - 1.16) | 0.95 (0.77 - 1.16) |
| Neglect x Time | 0.86 (0.73 - 1.01) | 0.86 (0.73 - 1.01) | 0.86 (0.69 - 1.06) | 0.86 (0.69 - 1.06) | 0.79 (0.62 - 1.00) | 0.79 (0.62 - 1.00) |
| Covariates |  |  |  |  |  |  |
| Male (ref. = female) | 2.38 (1.70 - 3.34)*** | 2.35 (1.68 - 3.30)*** | 1.84 (1.22 - 2.78)*** | 1.81 (1.20 - 2.73)*** | 5.44 (3.18 - 9.29) *** | 5.50 (3.21 - 9.42) *** |
| Middle school (ref. = primary school) | 5.21 (3.58 - 7.60)*** | 5.32 (3.65 - 7.76)*** | 5.91 (3.61 - 9.66)*** | 5.88 (3.59 - 9.62)*** | 10.76 (5.68 - 19.75) *** | 10.76 (5.68 - 19.75) *** |
| Parental absence (ref. = none) |  |  |  |  |  |  |
| One | 0.62 (0.39 - 0.96)* | 0.61 (0.39 - 0.95)* | 0.61 (0.36 - 1.04) | 0.61 (0.36 - 1.04) | 0.83 (0.44 - 1.50) | 0.81 (0.45 - 1.52) |
| Both | 0.96 (0.67 - 1.40) | 0.95 (0.65 - 1.37) | 0.62 (0.39 - 0.95)* | 0.62 (0.39 - 0.96)* | 0.86 (0.49 - 1.39) | 0.86 (0.51 - 1.44) |
| Subjective SES | 1.05 (0.89 – 1.24) | 1.05 (0.89 – 1.23) | 1.14 (0.94 – 1.39) | 1.14 (0.94 – 1.39) | 1.25 (1.01 - 1.58)* | 1.26 (1.01 - 1.58)* |
| School connection | 0.88 (0.73 - 1.07) | 0.87 (0.72 - 1.04) | 0.85 (0.66 - 1.08) | 0.81 (0.65 - 1.01) | 0.84 (0.63 - 1.12) | 0.84 (0.65 - 1.09) |
| Neighborhood connection | 0.95 (0.80 - 1.14) | 0.98 (0.81 - 1.19) | 0.90 (0.72 - 1.12) | 0.95 (0.74 - 1.21) | 0.90 (0.70 - 1.15) | 0.92 (0.69 - 1.31) |
| Abuse/neglect x School connection |  |  |  |  |  |  |
| EA x school connection | 1.20 (0.95 - 1.52) | - | 1.23 (0.93 - 1.63) | - | 1.00 (0.73 - 1.37) | - |
| PA x school connection | 0.80 (0.64 - 1.00) * | - | 0.82 (0.64 - 1.04) | - | 0.89 (0.68 - 1.16) | - |
| SA x school connection | 0.99 (0.81 - 1.22) | - | 0.97 (0.77 - 1.21) | - | 1.13 (0.88 - 1.45) | - |
| Neglect x school connection | 1.00 (0.81 - 1.23) | - | 0.92 (0.72 - 1.19) | - | 1.01 (0.76 - 1.34) | - |
| Abuse/neglect x Neighborhood connection |  |  |  |  |  |  |
| EA x NH connection | - | 1.24 (0.98 - 1.58) | - | 0.94 (0.71 - 1.25) | - | 0.95 (0.69 - 1.31) |
| PA x NH connection | - | 0.79 (0.60 - 0.98) * | - | 0.95 (0.73 - 1.24) | - | 0.98 (0.73 - 1.31) |
| SA x NH connection | - | 0.99 (0.80 - 1.23) | - | 1.01 (0.80 - 1.28) | - | 1.08 (0.83 - 1.40) |
| Neglect x NH connection | - | 0.90 (0.72 - 1.11) | - | 0.96 (0.74 - 1.25) | - | 0.95 (0.74 - 1.25) |

*Notes.* OR = odds ratios; 95% CI = 95% confidence intervals; SES = socioeconomic status; NH = neighborhood. *** *p* < .001, ** *p* < .01, * *p* <.05.

**Supplementary eTable 5.** Moderating effects of sex and grade in the association between childhood maltreatment and past-month drinking, binge drinking and smoking.

|  | Drinking |  | Binge drinking |  | Smoking |  |
| --- | --- | --- | --- | --- | --- | --- |
| Predictors | Sex Model | Grade Model | Sex Model | Grade Model | Sex Model | Grade Model |
| Time | 1.17 (1.03 - 1.33)* | 1.17 (1.03 - 1.33)* | 1.19 (1.00 - 1.43) | 1.19 (1.00 - 1.43) | 1.06(0.87 - 1.30) | 1.06(0.86 - 1.30) |
| Emotional abuse (EA) | 1.46 (0.94 - 2.25) | 1.21 (0.75 - 1.96) | 1.15 (0.68 - 1.93) | 1.17 (0.65 - 2.10) | 1.30(0.67 - 2.52) | 1.47(0.76 - 2.85) |
| Physical abuse (PA) | 0.71 (0.41 - 1.23) | 1.30 (0.84 - 2.01) | 1.00 (0.54 - 1.86) | 1.88 (1.12 - 3.14)* | 1.05(0.49 - 2.23) | 1.59(0.88 - 2.86) |
| Sexual abuse (SA) | 1.85 (1.16 - 2.93)* | 1.22 (0.83 - 1.79) | 2.17 (1.25 - 3.70)** | 1.23 (0.79 - 1.92) | 1.95(1.00 - 3.78)* | 1.24(0.75 - 2.06) |
| Neglect | 1.78 (1.17 - 2.70)** | 1.48 (0.97 - 2.27) | 1.95 (1.15 - 3.31)* | 1.65 (0.96 - 2.84) | 3.16(1.58 - 6.30)*** | 1.61(0.83 - 3.12) |
| Abuse/neglect x Time |  |  |  |  |  |  |
| EA x Time | 0.92 (0.77 - 1.11) | 0.92 (0.77 - 1.10) | 0.92 (0.73 - 1.16) | 0.92 (0.73 - 1.16) | 0.99(0.76 - 1.28) | 0.99(0.76 - 1.29) |
| PA x Time | 0.92 (0.76 - 1.10) | 0.92 (0.77 - 1.10) | 0.90 (0.73 - 1.11) | 0.91 (0.74 - 1.12) | 0.85(0.68 - 1.08) | 0.86(0.68 - 1.09) |
| SA x Time | 0.90 (0.77 - 1.05) | 0.89 (0.76 - 1.04) | 0.92 (0.76 - 1.10) | 0.90 (0.75 - 1.09) | 0.99(0.81 - 1.21) | 0.98(0.80 - 1.20) |
| Neglect x Time | 0.86 (0.73 - 1.01) | 0.86 (0.73 - 1.01) | 0.85 (0.69 - 1.05) | 0.86 (0.70 - 1.06) | 0.79(0.62 - 1.01) | 0.79(0.62 - 1.01) |
| Covariates |  |  |  |  |  |  |
| Male (ref. = female) | 2.56 (1.78 - 3.69)*** | 2.35 (1.68 - 3.30)*** | 1.94 (1.22 - 3.08)*** | 1.81 (1.20 - 2.72)*** | 8.04(4.13 - 15.64)*** | 5.74(3.33-9.90)*** |
| Middle school (ref. = primary school) | 5.17 (3.55 - 7.54)*** | 4.61 (3.03 - 7.01)*** | 5.85 (3.58 - 9.56)*** | 5.12 (2.87 - 9.15)*** | 10.58(5.78 - 19.38) | 8.34(4.02-17.30) *** |
| Parental absence (ref. = none) |  |  |  |  |  |  |
| One | 0.61 (0.39 - 0.96)* | 0.62 (0.39 - 0.97)** | 0.62 (0.36 - 1.05) | 0.61 (0.36 - 1.04) | 0.80(0.43 - 1.48) | 0.82(0.44 - 1.52) |
| Both | 0.95 (0.66 - 1.38) | 0.97 (0.67 - 1.41) | 0.61 (0.39 - 0.95)* | 0.63 (0.40 - 0.98)* | 0.82(0.49 - 1.38) | 0.85(0.50 - 1.42) |
| Subjective SES | 1.05 (0.89 – 1.24) | 1.05 (0.90 – 1.25) | 1.14 (0.93 - 1.38) | 1.15 (0.94 – 1.40) | 1.25(1.00 – 1.56)* | 1.25(1.00 – 1.56)* |
| School connection | 0.87 (0.72 - 1.04) | 0.86 (0.71 - 1.03) | 0.81 (0.65 - 1.01) | 0.80 (0.64 - 0.99)* | 0.84(0.65 - 1.08) | 0.82(0.64 - 1.06) |
| Neighborhood connection | 0.97 (0.81 - 1.16) | 0.96 (0.80 - 1.15) | 0.92 (0.73 - 1.14) | 0.90 (0.72 - 1.12) | 0.94(0.73 - 1.21) | 0.93(0.72 - 1.19) |
| Abuse/neglect x Sex |  |  |  |  |  |  |
| EA x Male | 0.75 (0.45 - 1.26) | - | 0.79(0.43 - 1.46) | - | 0.61(0.29 - 1.29) | - |
| PA x Male | 2.13 (1.16 - 3.90)** | - | 1.99(1.00 - 3.94) | - | 1.80(0.79 - 4.09) | - |
| SA x Male | 0.80 (0.48 - 1.33) | - | 0.87(0.48 - 1.56) | - | 0.95(0.46 - 1.92) | - |
| Neglect x Male | 0.73 (0.46 - 1.16) | - | 0.71(0.40 - 1.25) | - | 0.58(0.28 - 1.19) | - |
| Abuse/neglect x Grade |  |  |  |  |  |  |
| EA x Middle School | - | 1.06 (0.61 - 1.84) | - | 0.84 (0.43 - 1.62) | - | 0.52(0.25 - 1.11) |
| PA x Middle School | - | 0.94 (0.55 - 1.59) | - | 0.81 (0.44 - 1.48) | - | 1.04(0.53 - 2.06) |
| SA x Middle School | - | 1.59 (0.99 - 2.53) | - | 2.04 (1.19 - 3.48)** | - | 1.77(0.97 - 3.23) |
| Neglect x Middle School | - | 0.89 (0.55 - 1.43) | - | 0.82 (0.45 - 1.48) | - | 1.34(0.66 - 2.75) |

*Notes.* OR = odds ratios; 95% CI = 95% confidence intervals; SES = socioeconomic status. *** *p* < .001, ** *p* < .01, * *p* <.05.

**Supplementary eFigure 2**. Interaction effect between sex and physical abuse (PA) predicting past-month drinking (left) and interaction effect between grade and sexual abuse (SA) in predicting past-month binge drinking (right).
